# Surface mapping of gastric motor functions using MRI: a comparative study between humans and rats

**DOI:** 10.1152/ajpgi.00045.2024

**Published:** 2024-06-25

**Authors:** Xiaokai Wang, Fatimah Alkaabi, Minkyu Choi, Madeleine R. Di Natale, Ulrich M. Scheven, Douglas C. Noll, John B. Furness, Zhongming Liu

**Affiliations:** ^1^Department of Biomedical Engineering, University of Michigan, Ann Arbor, Michigan, United States; ^2^Division of Electrical and Computer Engineering, University of Michigan, Ann Arbor, Michigan, United States; ^3^Department of Anatomy and Physiology, University of Melbourne, Parkville, Victoria, Australia; ^4^Florey Institute of Neuroscience and Mental Health, Parkville, Victoria, Australia; ^5^Department of Mechanical Engineering, University of Michigan, Ann Arbor, Michigan, United States; ^6^Department of Radiology, University of Michigan, Ann Arbor, Michigan, United States

**Keywords:** diffeomorphic modeling, gastric accommodation, gastric emptying, gastric motility, peristalsis

## Abstract

The stomach’s ability to store, mix, propel, and empty its content requires highly coordinated motor functions. However, current diagnostic tools cannot simultaneously assess these motor processes. This study aimed to use magnetic resonance imaging (MRI) to map multifaceted gastric motor functions, including accommodation, tonic and peristaltic contractions, and emptying, through a single noninvasive experiment for both humans and rats. Ten humans and 10 Sprague-Dawley rats consumed MRI-visible semisolid meals and underwent MRI scans. We used a surface model to analyze MRI data, capturing the deformation of the stomach wall on ingestion or during digestion. We inferred muscle activity, mapped motor processes, parcellated the stomach into functional regions, and revealed cross-species distinctions. In humans, both the fundus and antrum distended postmeal, followed by sustained tonic contractions to regulate intragastric pressure. Peristaltic contractions initiated from the distal fundus, including three concurrent wavefronts oscillating at 3.3 cycles/min and traveling at 1.7 to 2.9 mm/s. These motor functions facilitated linear gastric emptying with a 61-min half-time. In contrast, rats exhibited peristalsis from the midcorpus, showing two wavefronts oscillating at 5.0 cycles/min and traveling at 0.4 to 0.9 mm/s. For both species, motility features allowed functional parcellation of the stomach along a midcorpus division. This study maps region- and species-specific gastric motor functions. We demonstrate the value of MRI with surface modeling in understanding gastric physiology and its potential to become a new standard for clinical and preclinical investigations of gastric disorders at both individual and group levels.

**NEW & NOTEWORTHY** A novel MRI technique can visualize how the stomach accommodates, mixes, and propels food for digestion in humans and animals alike. Digital models of gastric MRI reveal the functional maps, organization, and distinction of the stomach across individuals and species. This technique holds the unique potential to advance basic and clinical studies of functional gastric disorders.

## Graphical abstract

**Figure:**
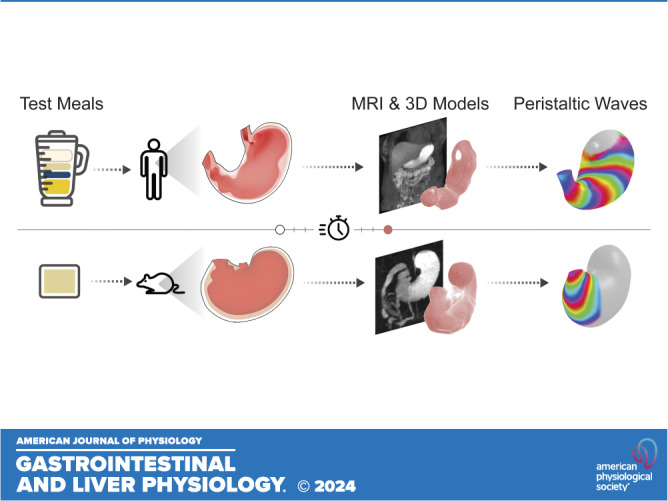

## INTRODUCTION

The stomach plays a crucial role in ingestion and digestion, driven by complex motor processes under neural and hormonal control (1–5). Upon consuming a meal, gastric muscles relax to accommodate food, engage slow tonic contractions to regulate intragastric pressure, and coordinate rapid peristaltic contractions for food breakdown and propulsion. In a healthy state, these processes are highly adaptive, variable, and coordinated across different regions, ensuring appropriate gastric emptying that depends on the size and content of the meal (2, 6). However, disruption of these processes can lead to gastric motor dysfunctions, manifesting as various symptoms (7–9), such as impaired gastric emptying or accommodation, nausea and vomiting, abdominal discomfort or pain, and inappropriate satiety. These symptoms are characteristics of many functional gastric disorders, recently grouped as disorders of gut-brain interaction (10, 11), such as gastroparesis (12, 13), functional dyspepsia (14–16), and dumping syndrome (17). Pinpointing how gastric motor processes contribute to individual symptoms and their underlying mechanisms is a significant challenge, partly due to the lack of a comprehensive diagnostic tool capable of characterizing gastric movements across various spatial and temporal scales in both health and disease (18). Hence, there is a pressing need for technical advancements in assessing gastric motor functions to improve diagnosis and inform treatments.

Current diagnostic tools, while useful, do not address all major aspects of gastric motor functions, and each has its own limitations (10, 18). For instance, gastric scintigraphy is the gold standard for measuring gastric emptying (19) but exposes patients to radiation and does not report gastric motility (19, 20). Breath tests provide a more convenient and nonradioactive way to measure gastric emptying but are less reliable (21, 22). Gastric barostat measures gastric accommodation but is invasive and does not report gastric emptying or motility (23, 24). Gastric manometry and endoscopy offer localized assessments (25, 26) but are invasive, lengthy, incomprehensive, and uncomfortable to patients (27). Electrogastrography measures gastric electrical activity with limited spatial resolution and specificity but does not report gastric emptying or accommodation and has limited clinical utility (10, 28, 29). Wireless motility capsules provide multifaceted readouts of whole gut transit (30, 31) but are unreliable and of low resolution regarding postprandial motility and emptying. To date, none of these existing methods can measure gastric movements with adequate resolution and specificity throughout the entire stomach. It is not feasible to use these tools simultaneously for combined assessments.

In contrast, gastrointestinal (GI) magnetic resonance imaging (MRI) provides unique opportunities for noninvasive, precise, and comprehensive analysis of gastric movements (32–38). Recent studies have used MRI-derived surface models to characterize gastric volume, tension (37), or motility (35, 36). However, those models were not standardized across sessions or individuals and thus unable to support group-level mapping of gastric functions. In contrast, we have developed a novel method to morph a surface template to match any realistic geometry of the rat stomach (39). This method enables us to characterize the kinematics of the stomach wall with high spatial and temporal resolution and to quantify and compare gastric motor functions both within and across individuals (39). In this study, we applied this approach to both humans and rats for a comparative analysis. This cross-species comparison is not only crucial for understanding the fundamental aspects of gastric motility, but also bridges the gap between preclinical and clinical studies to better inform clinical practices.

## MATERIALS AND METHODS

### Human Subjects and Study Design

**Figure 1.**
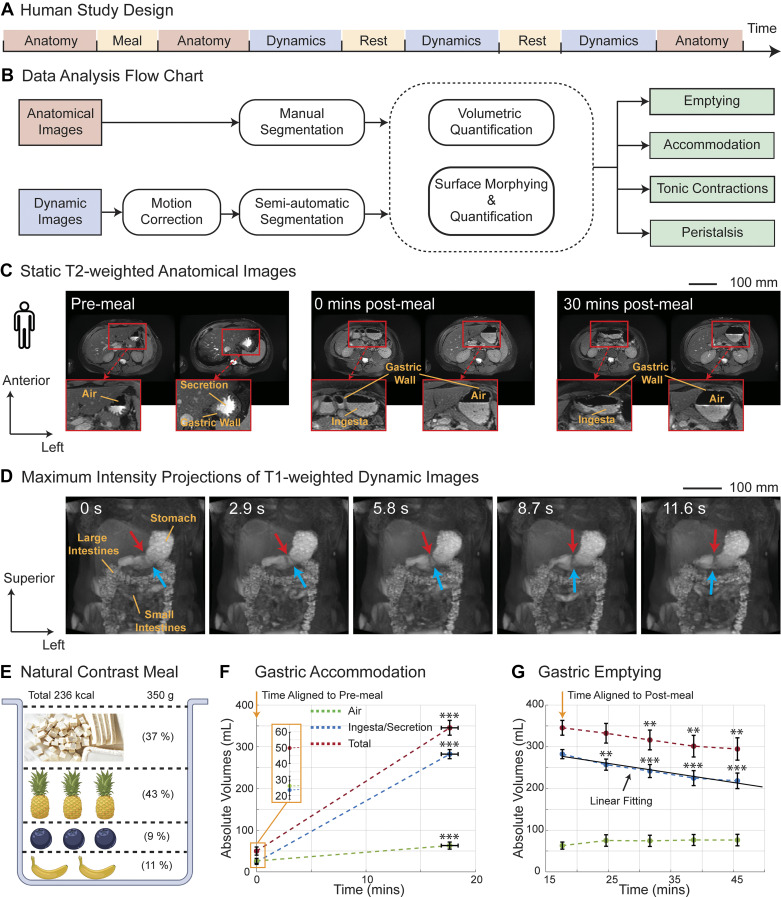
Human gastrointestinal (GI) MRI and volumetric quantifications. *A*: MRI protocol, including anatomical and dynamic scans and resting intervals. *B*: data analysis pipeline of anatomical and dynamic MRI images to quantify gastric accommodation, tonic and peristaltic contraction, and gastric emptying. *C*: T_2_-weighted anatomical scans before and after the meal. Labels highlight gastric walls and content (air, secretion, ingesta). *D*: T_1_-weighted dynamic scans at adjacent time points. Arrows highlight a contractile ring moving along the lesser (red) and greater (blue) curvatures. Labels indicate the stomach and small and large intestines. *E*: ingredients for the test meal: firm tofu, pineapple trunk and juices, blueberry, and banana. *F*: gastric volumes (air, ingesta/secretion, or total) before and after the meal indicate gastric accommodation. *G*: gastric volumes at different postmeal times indicate gastric emptying. Dark solid line shows the linear fitting. Error bars indicate SE. Paired single-side *t* tests were performed to test if the gastric volumes premeal are significantly lower than immediately postmeal in *F* and if the gastric volumes immediately postmeal are significantly higher than at a certain time postmeal in *G*. Statistical significance: **P < 0.01 and ***P < 0.001.

Ten healthy volunteers (6 females; age: 24.9 ± 1.0 yr; and body mass index: 23.1 ± 1.0, mean ± SE) participated in the study according to a protocol approved by the institutional review board at the University of Michigan. We excluded subjects who had abdominal surgery, were under prescribed medication, or were allergic to the ingredients of the contrast meal (36). Subjects deprived themselves of food, alcohol, and caffeine for ∼12 h overnight and of water for >1 h before the MRI in the morning. The study included a premeal scan as the baseline, followed by the consumption of a contrast meal and postprandial scans. During scans, subjects stayed in a 3-Tesla UHP MRI system (GE HealthCare, Chicago, IL) in a supine position with two flexible surface coils (AIR array coil; GE HealthCare) over both the anterior and posterior sides of the stomach. The contrast meal, including all-natural ingredients (Fig. 1*E*), was ∼350 g, 236 kcal (carbohydrate: 74%, protein: 13%, fat: 7%, and fiber: 6%). While holding their breath, each subject underwent T_2_-weighted two-dimensional (2-D) scans that detailed gastric anatomy and its morphological changes due to meal intake and emptying. With free breathing, each subject underwent multiple sessions of dynamic T_1_-weighted three-dimensional (3-D) MRI that captured the movement of the ingesta and stomach wall. Between dynamic sessions, a short break allowed subjects to rest briefly. The total scan time was ∼1 h. Figure 1*A* presents a summary of the study design.

### MRI Acquisition

The imaging protocol included anatomical scans with 2-D balanced steady-state free precession (FIESTA, repetition time (*TR*) = 3.42 ms, echo time (*TE*) = 1.35 ms, flip angle (FA) = 50^°^, field of view (FOV) = 400 × 400 mm^2^, matrix size = 192 × 300, 40–54 axial slices, acceleration = 2, axial slices, thickness = 3 mm, frequency encoding: anterior to posterior, and phase encoding: right to left). During the anatomical scan, subjects held their breath for 20 to 30 s. To capture fast dynamics, we acquired dynamic scans using a 3-D spoiled gradient echo with fat saturation under free breathing [LAVA, *TR* = 3.00 ms, *TE* = 1.32 ms, FA = 12°, FOV = 360 × 360 mm, coronal slab, thickness = 128–144 mm, and matrix size = 192 × 128 × (32–36), acceleration = 5, frequency encoding: superior to inferior, phase encoding: right to left] repeated at ∼3 s per volume for a total of 5 min. We slightly adjusted the acquisition parameters as needed to accommodate each subject’s body size and ensure full coverage of the stomach. For analysis, T_2_-weighted anatomical images were interpolated to 512 × 512 in-plane, and T_1_-weighted dynamic images were interpolated to 256 × 256 in-plane and by 2 in slice dimension.

### Data Analysis

We quantified the changes in intragastric volume to assess gastric accommodation and emptying and characterized the deformation and kinematics of the stomach wall to map regional gastric accommodation as well as tonic and peristaltic contractions, following the analysis pipeline shown in Fig. 1*B*.

#### Image processing.

We adapted our previously published approach to delineate the intragastric volume (36). Specifically, we manually segmented the intragastric volume and identified the stomach wall from the anatomical images. For dynamic scans, we first corrected for respiratory motion (36). Then, we averaged the motion-corrected images over time and manually segmented the time-averaged images. Furthermore, we segmented each frame through registration to the time-averaged images using a 3-D multiresolution symmetric diffeomorphic registration algorithm implemented by Advanced Normalization Tools (40). We ensured the quality of segmentation by visual inspection and corrected minor errors as needed. Note that the segmentation included intragastric air, in addition to the ingesta, to precisely delineate the total intragastric volume and its compartments.

#### Surface deformation.

Unique to our approach, we deformed a mesh-based stomach template (41) to enclose the total intragastric volume segmented from MRI scans. Using initial affine transformations and subsequent neural ordinary differential equations (42), we modeled how the stomach changed its shape across different states and times through diffeomorphic transformation, while the model complied with biomechanical constraints. As a result, the model-based morphing preserved the surface topology and precluded self-intersecting faces (39). The surface mesh, despite its complex morphing, remained a biomechanically plausible manifold. Based on the anatomical scans, the model captured progressive deformation of the stomach wall, indicative of gastric accommodation and tonic contractions. Based on the dynamic scans, the model captured the fast kinematics of the stomach wall, reflecting gastric motility.

#### Feature quantification.

We estimated the changes in intragastric volume before and after the meal, as well as during the postprandial period, to characterize gastric accommodation and emptying. This volumetric analysis was applied separately to gastric air, secretion or ingesta-secretion mix, and the total volume. In particular, we fitted a linear function to the postprandial changes of the ingesta-secretion mix to estimate the gastric emptying rate and half-time.

We inferred underlying muscle activity by characterizing surface deformation and kinematics. For gastric accommodation and tonic contractions, we assessed regional changes in the surface area. For more details about the analysis method, see appendix: *Surface Feature Quantification*. Gastric muscles, namely the longitudinal, circular, or oblique muscles, reside in different layers and along different orientations that vary with gastric regions (43, 44). Increases or decreases in surface area across states were quantified. These changes reflect the aggregated effects of the elongation or shortening of all types of muscle fibers, including muscle cell groups with different sizes, depths, and orientations in the stomach wall. These surface area measurements were obtained individually and then averaged across subjects. To reveal the spatially smooth deformations corresponding to cohesive muscle activity within regions, the group-averaged pattern was spatially smoothed through spectral decomposition (45) (see details in appendix: *Spatial Smoothing through Spectral Decomposition*). For more rapid phasic contractions, we estimated the displacement at each mesh vertex (surface point) every ∼3 s. The resulting surface deformation oscillated between inward and outward, reflecting contractile waves. To refine this complex pattern, we applied principal component analysis for noise reduction (46) (see details in appendix: *Noise Reduction through Principal Component Analysis*).

For a detailed multifaceted characterization of gastric motility, we examined the time series of point-wise displacement. For each vertex, we assessed the rhythmicity, frequency, and amplitude of the oscillation using the method described in Ref. 39. The rhythmicity, indicating the oscillatory nature of the movement, was evaluated as the ratio of the dominant oscillatory movement relative to the overall deformation; the frequency of the oscillation was defined as the frequency with the maximal power; and the amplitude of the oscillation was quantified as the peak-to-peak amplitude.

In addition, we identified the onset location from where gastric peristalsis was initiated. For different vertices in a distal-to-proximal direction along the greater curvature, we analyzed their phase differences relative to the most distal part of the antrum. This analysis could differentiate between areas with traveling waves, which show a progressive phase change, and areas with standing waves or no contractile waves, which exhibit constant or random phase differences. Then, we identified the surface points showing oscillatory contractions with zero phase differences relative to the onset zone at both the lesser curvature and greater curvature. Among them, we further identified the most proximal set that aligned themselves circumferentially to form a circular band spanning the lesser and greater curvatures. This circular band defined the “first wavefront” of peristalsis. Relative to this band, the distal areas were considered collectively as the “peristaltic pump” subject to subsequent analyses.

We evaluated motility features within the identified peristaltic pump, relevant to the organization, coordination, and speed of peristaltic waves. To map the spatial organization of peristalsis, we mapped the time-averaged relative phase difference between every vertex and the points that were previously identified with zero-phase differences relative to the onset zone. To characterize the coordination of peristalsis, we evaluated the phase-locking value (PLV) (47) between oscillatory contractions at different locations, averaged the PLV within the peristaltic pump, and referred it as the coordination index. The PLV is a time-averaged metric of synchronization between two oscillations, as widely used for assessing coordination in neuroelectric oscillations (48–50). Moreover, we measured the velocity of wave propagation along both the lesser curvature and the greater curvature, as the lower and upper bounds of the velocity throughout the stomach (see appendix: *Surface Feature Quantification* for further details). These motility features were first extracted for each subject and then averaged across subjects. The resulting group-level features were further refined by local averaging across direct spatial neighbors for noise reduction.

Using point-wise motility features, we parcellated the stomach into two functional regions, namely the proximal and distal stomach. Using *k*-means clustering, we grouped surface points into two clusters based on their affinity in space and similarity in functional features (39). These functionally determined regions were compared with the predefined anatomical regions: fundus, corpus, and antrum (44, 51). Additionally, we also examined variations in motility features, such as the frequency and amplitude, to discern differences between these data-driven functional regions and the conventionally defined anatomical regions.

#### Statistical analysis.

We tested the statistical significance of any difference in functional attributes between regions or conditions by using paired *t* tests with a significance level of 0.05 or lower. Outliers were detected for each region or condition if a value was out of distribution, being greater than the 75th percentile or lower than the 25th percentile by 1.5 times the interquartile range. Outliers were highlighted in box or violin plots for visual inspection but not excluded from significance tests. Additionally, we used an *F* test at a confidence level of 99.9% to assess the linear model for gastric emptying.

### Animal Study Design

For comparison, we used similar acquisition and analysis methods to image and map gastric peristalsis in rats. Specifically, we used 10 Sprague-Dawley rats (male: 250–350 g; Envigo, Indianapolis, IN), following procedures approved by the Unit for Laboratory Animal Medicine and the Institutional Animal Care and Use Committee at the University of Michigan. After 1 week of diet training (see details in Ref. 39), every rat was able to voluntarily consume ∼5 g Gd-DTPA-labeled dietgel 15–30 min before the MRI. We then anesthetized the rats initially with 4% isoflurane, then 2.5% isoflurane, followed by a subcutaneous bolus injection of dexmedetomidine (dex; Zoetis, Parsippany, NJ; 0.01 mg/mL, 0.0125 mg/kg) during scan preparation, and then subcutaneous infusion of dex (0.01 mg/mL, 0.025 mg/kg/h) alongside <0.5% isoflurane delivered through facemask during the entire imaging time. We constantly monitored vital signs throughout the experiment and adjusted the anesthesia as needed to maintain a stable physiological condition.

We imaged the GI tract using a 7-Tesla small-animal MRI scanner (Varian; Agilent Technologies, CA). The imaging protocol consisted of repeated 2-D multislice gradient echo sequences with respiratory gating (*TR* = 10.50 ms, *TE* = 1.44 ms, FA = 25°, FOV = 64 mm × 42 mm, matrix size = 128 × 84, slice thickness = 1.5 mm, 20 slices, and temporal resolution: < 3 s) for a total time of ∼4 h (see details in Ref. 39). To leverage the rich information in rat MRI, we conducted the same surface-based analysis with a rat stomach template (52), in parallel to human data, for cross-species comparison. See details in *Data Analysis*.

## RESULTS

We employed a novel approach to map gastric motor functions in both healthy humans (*n* = 10) and rats (*n* = 10). Using MRI, we imaged the ingested content and its movement within the GI tract after subjects consumed a contrast-labeled test meal (Fig. 1, *A*, *C*, and *D*). Through surface modeling, we tracked the movement of the stomach wall and generated functional maps of gastric accommodation, tonic and peristaltic contractions, and emptying. These maps, represented on a species-specific surface template, provided a basis for a holistic understanding of normal gastric motor functions and comparisons within and between species.

### Human MRI Visualizing Gastric Anatomy and Dynamics

For human subjects, T_2_-weighted anatomical MRI provided high-resolution images of the intragastric content and the stomach wall before and after meals and during digestion (Fig. 1*C*). After overnight fasting, the stomach had a small volume (50 ± 10 mL, mean ±SE evaluated across subjects), enclosing both air (26 ± 6 mL) and secretion (24 ± 6 mL). After the subjects consumed a smoothie-like meal with all-natural ingredients (Fig. 1*E*), the stomach expanded its volume to 345 ± 18 mL for storing the ingested meal (237 ± 10 g) plus gastric secretions, with a minor increase of the air volume (Fig. 1*F*). During digestion, the volume of the ingesta gradually decreased, showing a linear trend of emptying, by 23 ± 5% at ∼28 min postmeal with an estimated half-emptying time of 61 min (Fig. 1*G*). Note that the stomach wall itself was also visible with MRI (Fig. 1*C*). Before the meal, the gastric wall appeared dark gray in contrast to intragastric content and adjacent organs like the liver. Prominent mucosal folds were also visible at this time. After the meal, the stomach wall became dimmer and thinner due to muscle elongation for gastric accommodation.

In addition, T_1_-weighted MRI scans, repeated at ∼3-s intervals, captured the movement of ingesta within the stomach (Fig. 1*D*). The ingesta appeared bright due to contrast enhancement by the T_1_-shortening manganese ions in the natural meal. The dynamic scans showed the ingesta being propelled through the propagation of muscle contractions. These contractions were more “occlusive” toward the antrum, forming dark bands in the maximal intensity projections onto the coronal plane. The contractions propagated down the stomach to facilitate mixing, grinding, and propulsion.

### Surface Mapping of Human Gastric Motor Functions

**Figure 2.**
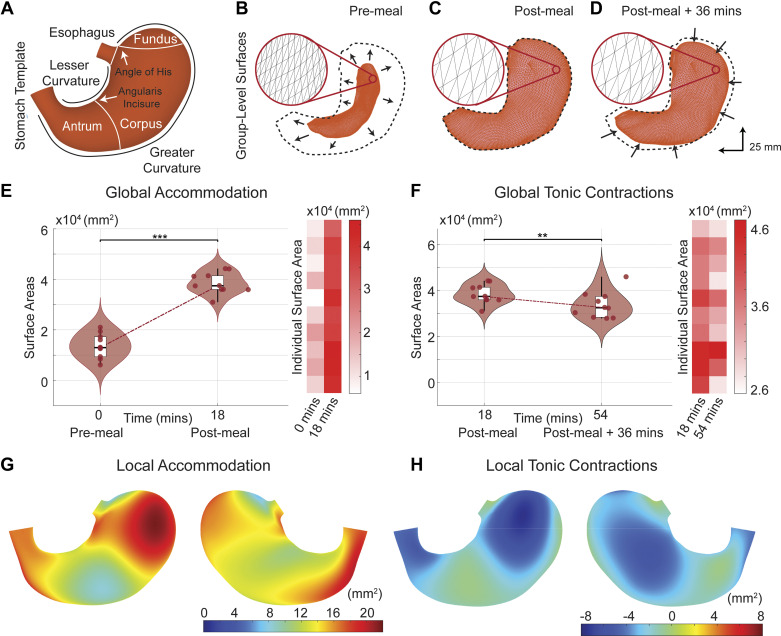
Human gastric accommodation and tonic contraction. *A*: surface template of the human stomach with anatomical labeling. *B*–*D*: surface morphology of the stomach right before, right after, and about 36 min after the meal, respectively. *Insets*: zoom of an area showing the changing lengths of edges and areas of surface elements, indicating local changes in the mesh model. Dashed lines illustrate the stomach’s shape right after the meal, as a reference for comparisons. Arrows indicate the trend of deformation from before to after the meal for gastric accommodation or from the early to later stage of digestion due to tonic contractions. *E* and *F*: changes of the total surface area as the overall measures of gastric accommodation and tonic contractions, respectively, for each individual (red dots and heatmaps) and group statistics (violin and box plots). Time was calibrated to the premeal baseline (*time 0*). The variation of the red dots along the *x*-axis shows the variation in the calibrated time across individuals. The box plot shows the median, 25th and 75th percentiles, and the violin plots show the distribution estimated from individual data. Paired single-sided *t* tests were performed to test if the gastric surfaces premeal are significantly lower than immediately postmeal in *E* and if the gastric surfaces immediately postmeal are significantly higher than 36 mins postmeal in *F*. Statistical significance: *P < 0.05, **P < 0.01, and ***P < 0.001). *G* and *H*: surface mapping of local areal expansion or shrinkage due to gastric accommodation or tonic contractions, respectively.

We attributed the observed changes in intragastric volume to the deformation of the stomach wall driven by gastric muscle elongation, tonic, and peristaltic contractions, or relaxations. To study these motor processes, we morphed a surface mesh model of the stomach from a generic shape (Fig. 2*A*) to fit the intragastric volumes segmented from MRI scans (39). The deformed surfaces captured both the morphology and kinematics of the stomach wall, providing surrogate measurements of various gastric motor functions.

Upon meal ingestion, we observed notable changes in the stomach’s morphology (Fig. 2, *B* and *C*). The ingested meal caused a considerable increase in the total surface area from 136 ± 15 cm^2^ before the meal to 385 ± 13 cm^2^ afterward (Fig. 2*E*). The local expansion was uneven across different regions. The ventrolateral fundus and distal antrum along the greater curvature showed the greatest expansion, whereas the corpus showed less expansion (Fig. 2*G*). These results demonstrate the stomach’s ability in flexible reshaping and suggest the specific regions, i.e., the ventrolateral fundus and distal antrum, responsible for gastric accommodation and the early stage of digestion.

As digestion proceeded, we observed a gradual decrease in the stomach’s total surface area, amounting to an average reduction of 13% by 37 min after the meal (Fig. 2*F*) as a result of tonic contractions. As quantified by local area shrinkage, the effects of tonic contractions were pronounced in the ventral side of the fundus and the distal end of the antrum (Fig. 2*H*). In contrast, the middle portion of the stomach, mainly the corpus, showed a slight expansion indicative of muscle elongation (Fig. 2*H*). These results illustrate the differential roles of various regions in establishing the stomach as a “pressure pump,” which regulates intragastric pressure gradients to facilitate digestion.

**Figure 3.**
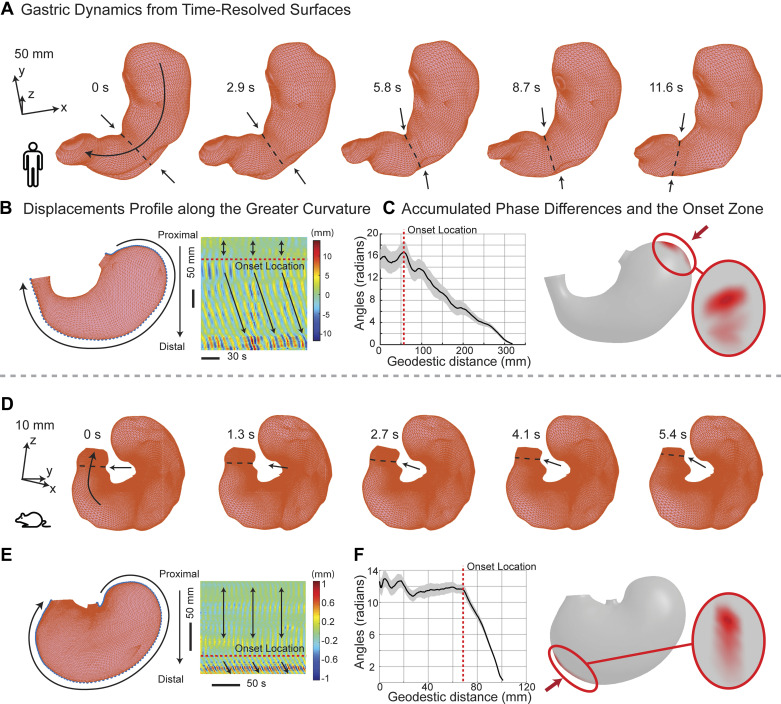
Gastric motility in humans and rats. *A* and *D*: dynamic changes of the surface morphology for human and rat stomachs, respectively. Arrows indicate a circumferential contractile wavefront (dashed line) traveling in time and in space. *B* and *E*: displacements of individual surface points along the greater curvature as a function of time (*x*-axis) and space (*y*-axis in a proximal to distal direction). Negative or positive displacements indicate inward or outward movements, respectively. Red dashed lines indicate the peristalsis onset location, which separates standing waves (proximal region, bidirectional dark arrows) and traveling waves (distal region, unidirectional dark arrows). These results are based on data from a representative human or rat subject. *D* and *F*: illustration of the peristalsis onset location based on the phase progression (dark line, mean; gray zone, SE) relative to the distal end. *C* and *F*, *right*: summarize the onset zones across individuals for both species.

In addition to tonic contractions, surface modeling of dynamic MRI captured peristaltic waves. These waves manifested as circumferential wavefronts, mainly traveling from the proximal to the distal direction (Fig. 3*A*). Our initial analysis, focusing on surface points along the greater curvature (Fig. 3*B*), revealed the initiation and propagation of this traveling wave (Fig. 3, *B* and *C*). Proximal points exhibited weaker standing waves, while more distal points exhibited stronger traveling waves (Fig. 3, *A* and *B*). This observation suggests the amplification and progression of phasic contractions down the stomach. Furthermore, we determined the onset site of peristalsis by identifying the area of transition, along the greater curvature, from the standing to traveling waves featuring a progressive phase shift (Fig. 3*C*). The onset zone summarized over all individuals showed that the peristaltic contraction waves originated from the distal fundus (Fig. 3*C*). The speeds of peristalsis were 2.91 ± 0.29 mm/s along the greater curvature and 1.70 ± 0.10 mm/s along the lesser curvature. These results highlight the stomach’s role as a “peristaltic pump,” which mixes, grinds, and propels the ingested meal through coordinated motility.

**Figure 4.**
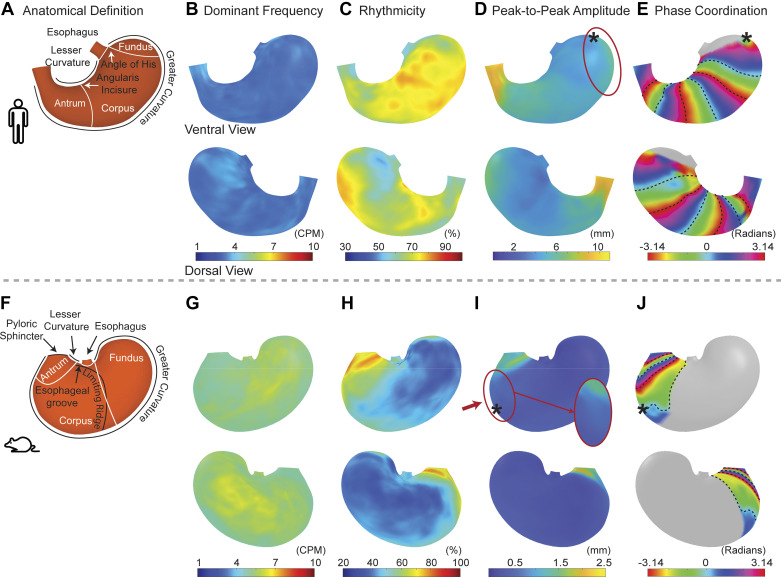
Features of gastric motility in humans and rats. *A* and *F*: illustrate human and rat stomachs with anatomical labeling. For each surface location, the presented features include the dominant frequency (*B* and *G*), the relative strength of the contractions at the dominant frequency, namely rhythmicity (*C* and *H*), peak-to-peak amplitude (*D* and *I*), and relative phase differences (*E* and *J*). *Peristalsis onset zones. *Insets*: zoom of an area around the onset. Relative phase maps reveal the spatial organization of peristalsis, showing 3 wavefronts in humans and 2 wavefronts in rats, with each 0 and ±π marked by a dashed line.

We further mapped the characteristics of peristaltic contractions for the group of human subjects (Fig. 4). The frequency of peristaltic contractions was largely consistent throughout the stomach, averaging 3.31 ± 0.08 cycles per minute (cycles/min). We observed a gradient (Fig. 4*D*) in peak-to-peak amplitude, with the antrum showing the strongest contractions (7.46 ± 0.47 mm), in contrast to the fundus (4.81 ± 0.39 mm) and corpus (4.67 ± 0.22 mm). The distal fundus, marking where the stomach initiated its function as a peristaltic pump, displayed stronger contractions than adjacent regions. Mapping of the phase differences from the onset zone revealed the spatial organization and temporal coordination of peristaltic waves. The coordination index of these waves, quantified as the PLV (47) averaged across locations within the peristaltic pump, was 0.68 ± 0.03 (in a range from 0 to 1), indicative of a well-coordinated peristaltic mechanism in a healthy stomach.

**Figure 5.**
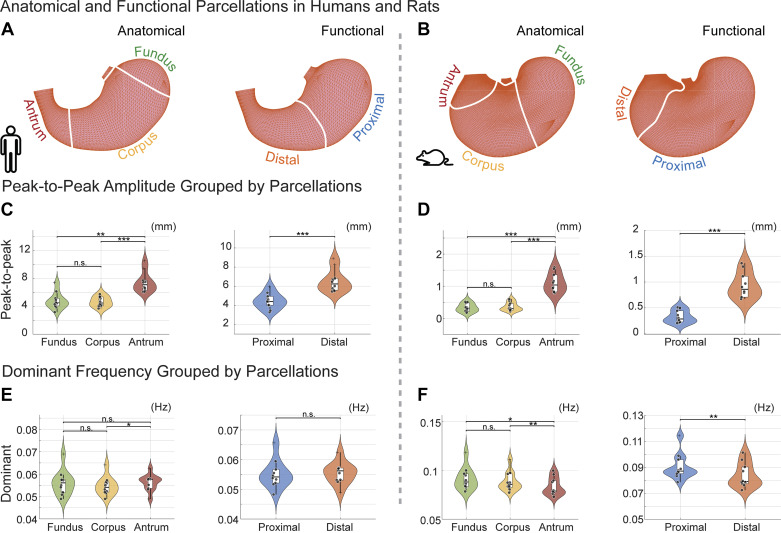
Motility differences across anatomical and functional gastric regions in humans and rats. *A* and *B*: anatomical vs. functional regions. *C*–*F*: peak-to-peak amplitude (*C* and *D*) and dominant frequency (*E* and *F*) are compared across anatomical or functional regions based on data from all subjects per species. Dark dots show individual results with random jitters. Violin and box plots show the variation across individuals. Plus signs indicate detected outliers that are either 1.5 the interquartile ranges above the 75th percentile or 1.5 the interquartile ranges below the 25th percentile. *C*–*F*: paired two-sided *t* tests were performed to test the statistical significance of the peak-to-peak amplitude (*C* and *D*) or dominant frequency (*E* and *F*) among different compartments. Outliers are included in the statistical test. Statistical significance between regions: *P < 0.05, **P < 0.01, and ***P < 0.001; n.s., not significant.

The regional distinctions in gastric motor activities did not align exactly with the anatomically defined regions: the fundus, corpus, and antrum, as has been recently pointed out (44). Based on the imaging evidence and feature quantification, we parcellated the stomach into two regions, namely the proximal and distal stomach (Fig. 5*A*), following the common terminology (44). As a result, the proximal stomach, including the fundus and the proximal corpus, exhibited contractions with an average amplitude of 4.45 ± 0.26 mm and a dominant frequency of 3.31 ± 0.09 cycles/min. The distal stomach, including the distal corpus and the antrum, exhibited contractions with an average amplitude of 6.55 ± 0.37 mm and a dominant frequency of 3.32 ± 0.07 cycles/min. The functional distinction between the proximal and distal stomach was more evident in the amplitude (Fig. 5*C*, paired *t* test, *P* < 0.001), rather than the frequency (Fig. 5*E*, paired *t* test, *P* = 0.64).

### Cross-Species Comparison

**Figure 6.**
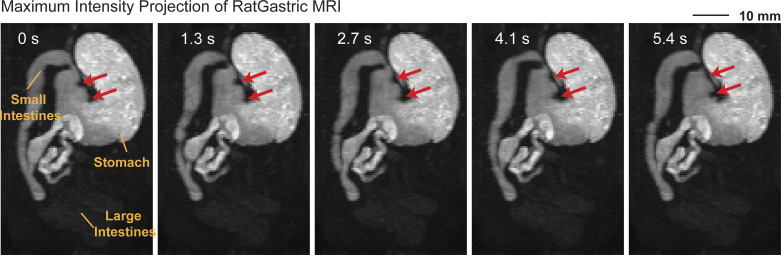
Dynamic gastroinestinal (GI)-MRI in rats. Images shown are maximal intensity projections onto an oblique plane at different time points separated by ∼1.3 s. Arrows indicate contractile wavefronts along the lesser curvature. Labels indicate GI compartments.

Similarly, applying this analysis to rats, we explored both similarities and differences in gastric peristalsis between rats and humans. After the consumption of a Gd-labeled meal (∼5 g), fast T_1_-weighted MRI scans in anesthetized rats revealed the intragastric content and its movement (Fig. 6). Surface analysis captured movements of the gastric wall given muscle contractions and relaxations (Fig. 3*D*). The peak-to-peak amplitude varied significantly and increased progressively (Fig. 4*I*) across the fundus (0.33 ± 0.04 mm), corpus (0.36 ± 0.04 mm), and antrum (1.11 ± 0.09 mm). However, the frequency of contraction showed a different trend: the fundus (5.49 ± 0.22 cycles/min), corpus (5.33 ± 0.19 cycles/min), and antrum (5.02 ± 0.17 cycles/min). These functional distinctions did not fully align with anatomical definitions of regions in rats (44). Similar to humans, we functionally parcellated the rat stomach into proximal and distal parts, as illustrated in Fig. 5, *B*, *D*, and *F*, highlighting the difference between anatomical and functional regions.

It is worth noting that the onset zone of peristalsis was around the midcorpus (Fig. 3*F*), rather than the distal fundus in humans (Fig. 3*C*). Proximal to this onset zone, minimal peristaltic activity was observed (Fig. 4*H*), while beyond it, two peristaltic waves were detected at the same time (Fig. 4*J*), moving toward the pylorus at speeds of 0.92 ± 0.08 mm/s and 0.35 ± 0.03 mm/s along the greater and lesser curvatures, respectively. These waves in rats were highly coordinated with a coordination index of 0.70 ± 0.04, comparable to humans. In summary, humans and rats exhibit notable differences in the location (Fig. 3) and scope (Fig. 4) of peristaltic initiation and activity, whereas both species exhibited a similar nature of highly coordinated gastric motility.

## DISCUSSION

In this work, we leveraged our latest advancement in MRI acquisition and analysis (34, 36, 39, 53, 54) for both preclinical and clinical studies of gastric motor function. Our approach enabled quantitative, simultaneous, and holistic assessment of gastric accommodation, tonic and peristaltic contractions, and emptying, resulting in functional maps relevant to how the stomach functions as a food reservoir, a pressure pump, and a peristaltic pump. Representing these maps on a surface template allowed us to average and compare gastric motor functions within and between individuals and species. Results demonstrate the potential of dynamic gastric MRI to become the next gold standard for studying gastric physiology and disorders.

Our data provide insights into normal gastric accommodation in response to a semisolid, smoothie-like test meal in humans. This accommodation manifests as notable surface expansion of the fundus as well as the antrum, likely due to the active elongation and stretch of gastric muscles in these regions, in line with prior work (37). The expansion of the fundus, known as the primary compartment for accommodation (2), is expected to facilitate food storage during meal consumption. Interestingly, the antrum, which traditionally is not considered to be involved in accommodation, also distends significantly, in line with the findings from a recent study (55). A plausible reason is that the liquid component of the ingested food quickly moves into the antrum through a gastric tunnel (Magenstrasse) along the lesser curvature (56), integrating with early digestion. Moreover, spatial patterns are not fully symmetric between the ventral and dorsal stomach, likely due to the supine position of subjects lying inside the scanner (57) and the compliance of adjacent structures, e.g., the liver and diaphragm being relatively solid.

This study refines our understanding of how the stomach functions as a pressure pump. Our results suggest that slow tonic contractions, characterized by the shortening of gastric muscles and the regional shrinkage of surface area, occur in both the fundus and the antrum. Effectively, these two regions regulate the intragastric pressure from both the proximal and distal ends of the stomach. This dual regulation creates a flexible system for managing the pressure gradient within the stomach. Higher proximal pressure aids anterograde movement of the content, while higher distal pressure aids retrograde movement (58). This coordination allows the stomach to propel or grind the food (59) and ensures a proper rate of emptying that depends on the size and content of the meal (2, 6). As this coordination spans over a long axial distance, it likely involves extrinsic pathways of gut-brain interaction (4, 60) rather than intrinsic circuits alone, which have relatively more localized spatial effects (51).

Our data also uncover new details about peristaltic contractions and their distinctions between rats and humans. In humans, peristaltic contractions span a long distance, covering nearly the entire stomach. This challenges the conventional view that peristaltic contractions are confined to the antrum and the distal corpus (2). Our data pinpoint the onset zone of peristaltic contractions to the distal fundus, which sets apart distinct patterns of contractile activities. Initiated from the onset zone, contractions travel in an orderly fashion and exhibit increasing amplitudes toward the pylorus. Proximal to the onset zone, weaker phasic contractions occur as a standing wave in the uppermost part of the fundus. Relevant to this finding, a prior study also suggests that the fundus is not fully quiescent; instead, it shows oscillating electrical activity around 5 cycles/min in humans (61). The onset zone of peristalsis is likely close to the pacemaker region of slow waves that are generated and propagated by interstitial cells of Cajal (ICC) (62). However, the peristalsis onset zone identified with MRI appears to be more proximal than that of the slow wave, which was previously identified with extracellular recordings in humans (63). This distinction between electrical and mechanical waves awaits further confirmation and investigation, partially because extracellular recordings entangle contributions from ICC, and muscle cells, among others (64).

The patterns of peristaltic contractions are generally similar between rats and humans. Yet, a notable distinction is that peristaltic contractions in the rat stomach are more confined, with the onset zone located at the midcorpus, much more distal than it is in humans. With respect to this onset zone, phasic contractions are minimal at proximal locations but strong and coordinated at distal locations. Unlike peristalsis in the small and large intestines, generated through the enteric nervous system, initiation of gastric peristalsis and its conduction are not neurogenic (65, 66). In the stomach, peristalsis is dependent on the electrical pacemaking activity of myenteric ICC, of which the electrical activity is conducted to the circular muscle through intramuscular ICC (67), giving rise to the circumferential contractions that are conducted from the onset zone to the pylorus. These distinctions in the patterns of peristaltic contraction may reflect cross-species differences in the distributions and connections of various cell types (44), especially ICC, although precise interpretation may be confounded by the effects of anesthesia (68, 69), which we used for rats but not humans in this study. To avoid this confound, it is desirable to perform GI MRI with conscious rats, as previously done for functional MRI (70, 71). This would involve behavioral training to acclimate rats to the MRI environments and necessitate refinements in image acquisition and analysis to deal with body motion due to respiration. Nevertheless, we anticipate the MRI-based comparatives between rats and humans can help translate findings from rats to humans.

Our study demonstrates the unique value of using a deformable and generic surface template to model the stomach (39). This leverages an algorithm for diffeomorphic transformation of a generic mesh of the stomach to match the changing geometry of the stomach across times, states, or individuals, as originally described for rats in Ref. 39 and demonstrated for humans in this study. Uniquely, the algorithm ensures that the deformed mesh preserves the topology of the original mesh. Their vertices follow the same ordering and grouping into faces. Vertices and faces remain at their same relative positions as the mesh deforms to match stomach shapes with large variations in size at fasted and fed states (Supplemental Video S1) and in individuals (Supplemental Fig. S1), or dynamic stomach shapes across different times due to muscle activity (Supplemental Video S2). The algorithm uses affine transformation, neural ordinary differential equations, and an L2-norm edge regularizer (effectively imposing a constraint on the global strain energy), all of which constrain the deformed mesh to remain smooth, while precluding intersecting faces or dramatic variation of edge lengths during deformation. Inherent to our algorithmic design, these constraints ensure that the mesh spans a topology-preserved manifold, describing the complex and variable geometry of the stomach within biomechanically plausible limits.

This approach provides a standard way for registering MRI-derived features to a surface template, bypassing the challenges posed by a large range of variations in the size, shape, and movement of the stomach. It supports both individual and group-level mapping of gastric morphology and dynamics with high spatial specificity. Although the strategy of using a surface template is common for studies of other organs, especially for human brain mapping (72, 73), its application to mapping gastric motor function is novel. We anticipate that this technique will merit broader applications with more subjects, diverse cohorts, and various diseases. Such applications will not only establish population-level baseline maps of normal gastric motor function and characterize the effects of biological variables and demographic factors but also enable patient-specific maps of dysfunctions for precision medicine in gastroenterology. Compared with existing techniques in probing gastric functions, our technique is more comprehensive, revealing how multiple aspects of motor functions collectively contribute to digestion under physiological and potentially pathophysiological conditions. As MRI is noninvasive, it may be used to monitor disease progression and evaluate treatment efficacy. Standardized analysis, as described in this paper, may motivate future efforts to acquire and curate more data to fuel medical artificial intelligence for evidence-based diagnosis of GI diseases. We anticipate that this technique will provide a promising pathway for the standardization and curation of gastric MRI data, facilitating the development of artificial intelligence-assisted clinical applications.

This study presents a holistic view of gastric motor functions, incorporating assessments of gastric accommodation, tonic and peristaltic contractions, and gastric emptying. Collectively, quantitative features of these motor functions enable data-driven parcellation of the stomach. At the simplest level, we divide the stomach into two functional parts: the proximal and distal stomach, with their division occurring around the midcorpus. As recently highlighted (44), the so-defined functional regions are different from anatomical regions and likely include subdivisions. In addition, the dynamic mesh model can be used for computational fluid dynamics simulations (58, 74), which can help further elucidate and differentiate the contributions of gastric motility and intragastric pressure to gastric emptying.

We acknowledge some limitations of our study. First, our analysis does not address the pylorus. Pyloric opening and closing coordinate with antral and duodenal contractions to regulate the rate of gastric emptying (2, 53, 75). MRI can potentially visualize and characterize this dynamic process (53). For reliable analysis of the dynamics of the pyloric sphincter and the antro-pyloro-duodenal coordination, pushing MRI acquisition with higher spatial and temporal resolution would be beneficial. Second, our study does not consider gastric secretion or release of acids, enzymes, or hormones, which may influence gastric motility. Given the feasibility of using MRI to detect gastric secretion (76, 77), future studies could aim to integrate multiple MRI acquisition protocols for a more comprehensive assessment of gastric functions. Third, our model approximated the stomach as a 2-D surface in the 3-D space and ignored the thickness of the stomach wall or variations in its individual layers. This model simplifies our analysis for mapping gastric motor functions across individuals and species. This simplification is a sensible choice since the gastric wall thickness is significantly smaller than the intragastric luminal volume in most postprandial conditions. However, a 3-D model considering the layered structure of the stomach wall would likely be more biologically accurate (43, 78). Gastric muscles reside in different layers, align in different orientations depending on gastric region, and receive different types of neural innervation. It will be desirable in the future to develop and validate methods to build 3-D stomach models that can explicitly account for different muscle layers, separate deformation patterns by layer orientation, and support more specific interpretations in terms of the strain, stretch, or length of underlying muscle fibers.

## DATA AVAILABILITY

Data will be made available upon reasonable request.

## SUPPLEMENTAL MATERIAL

10.6084/m9.figshare.25918021.v2 Supplemental Video S1: https://doi.org/10.6084/m9.figshare.25918021.v2.

10.6084/m9.figshare.25918054.v1 Supplemental Video S2: https://doi.org/10.6084/m9.figshare.25918054.v1.

10.6084/m9.figshare.26009002 Supplemental Fig. S1: https://doi.org/10.6084/m9.figshare.26009002.

## GRANTS

This work was supported by National Institutes of Health Grants DK131524, AT011665, OD030538, EB034344, and OD023847 and by the University of Michigan.

## DISCLOSURES

No conflicts of interest, financial or otherwise, are declared by the authors.

## AUTHOR CONTRIBUTIONS

X.W. and Z.L. conceived and designed research; X.W., F.A., U.M.S., D.C.N., and Z.L. performed experiments; X.W. analyzed data; X.W., F.A., M.C., M.R.D.N., J.B.F., and Z.L. interpreted results of experiments; X.W. prepared figures; X.W. and Z.L. drafted manuscript; X.W., F.A., M.C., M.R.D.N., U.M.S., D.C.N., J.B.F., and Z.L. edited and revised manuscript; X.W., F.A., M.C., M.R.D.N., U.M.S., D.C.N., J.B.F., and Z.L. approved final version of manuscript.

## APPENDIX

### Spatial Smoothing through Spectral Decomposition

We assume that muscle activity should be cohesive within local regions. We applied spatial smoothing to the estimated changes in local surface area across different states (e.g., pre- vs. postprandial) to eliminate processing artifacts and highlight the regional distributions of local effects. Specifically, spatial smoothing used spectral decomposition of the surface mesh, structured as an undirected graph of *N* vertices (or nodes) interconnected via edges. The equation below expresses the Laplacian matrix ($L\in R^{N\times N}$) of the graph in terms of its spectral decomposition by eigenvectors ($E\in R^{N\times N}$) and eigenvalues ($\Lambda \in R^{N\times N}$).

L=EΛE′

where ′ denotes the transpose of a matrix. For a feature vector $\alpha \in R^{N\times 1}$, we projected it to the subspace spanned by the first *S* = 20 eigenvectors, through multiplication with the matrix ($E_{S}\in R^{N\times S}$). As the eigenvectors were ordered to have increasingly higher spatial frequencies, this equation below effectively acted as a spatial low-pass filter, resulting in a spatially smoothed feature vector ($\alpha_{S}\in R^{N\times 1}$).

$$\alpha_{S}=E_{S}E_{S}\prime \alpha .$$

The hyperparameter *S* was determined by visual inspection as a heuristic choice to favor intersubject alignment while avoiding oversmoothing.

### Noise Reduction through Principal Component Analysis

We performed noise reduction using principal component analysis (low-rank approximation) on dynamic displacements of the stomach surface points before extracting motility features. From dynamic scans, we represented the displacements of all vertices (surface points) over time as a matrix ($D\in R^{N\times T}$), where *N* is the total number of vertices and *T* is the total number of time points (*T* < *N*). We then performed singular value decomposition on the matrix *D* as

D=UΣV′,

where $U\in R^{N\times T}$, $\Sigma \in R^{T\times T}$, and $V\in R^{T\times T}$. We reconstructed the denoised low-rank version of the matrix ($D_{P}\in R^{N\times T}$),

$$D_{P}=U_{P}\Sigma_{P}V_{P}\prime ,$$

where $U_{P}\in R^{N\times P}$, $\Sigma_{P}\in R^{P\times P}$, and $V_{P}\in R^{T\times P}$. We chose the hyperparameter *P* = 10 so that the displacements were temporally smoothed while not introducing additional patterns.

### Surface Feature Quantification

Based on the derived stomach surfaces from anatomical scans, we estimated the sustained effects of gastric accommodation and tonic contractions on local surface geometry, through evaluating the changes in the local surface area surrounding each vertex. The stomach surface is a watertight triangulated mesh. Per each vertex, we summed the areas of all faces that included that vertex, and evaluated the vertex-wise areal changes across states. We visualized the spatial pattern of the changes in local surface geometry after spatial smoothing, as described in *Spatial Smoothing through Spectral Decomposition*.

Based on the derived stomach surfaces from dynamic scans, we defined and quantified the frequency, amplitude, and rhythmicity of the oscillations (displacements), using the method previously described (39). These features were calculated for each vertex based on its estimated displacement from its time-averaged location within a session. The magnitude of the displacement was the Euclidean distance between its instantaneous 3-D position and the time-averaged 3-D position, whereas the sign of the displacement was positive (negative) if the displacement was outward (inward), indicating muscle relaxation (contraction). We further performed noise reduction on the displacements before feature quantification, as described in *Noise Reduction through Principal Component Analysis*. Additionally, given the estimated onset zone, we defined the peristaltic pump of the stomach and quantified the propagation velocity, spatial phase coordination, and coordination index of the gastric peristalsis within the pump. These features were calculated from the estimated displacements after noise reduction and bandpass filtering around the identified dominant frequency of peristalsis. All features were averaged across all sessions and subjects to generate group-level estimations. We provide more details of the quantification methods for each defined quantity in the following subsections.

### Frequency, Amplitude, and Rhythmicity

#### Frequency.

The frequency of the oscillation was defined as the frequency with the maximal power. Specifically, given the estimated vertex-wise displacement in each session, we estimated its power spectral density to identify the frequency that had the largest power within the frequency range of 0.1–6 cycles/min in humans and 0.1–10 cycles/min in rats.

#### Amplitude.

The amplitude of the oscillation was defined as the peak-to-peak amplitude. For a time series of displacements per vertex (the vertex was indexed as *p*), we identified the maximal values [positive, relaxations, *a*_1_(*p*, *i*)] and minimal values [negative, contractions, *a*_2_(*p*, *i*)] per relaxation-contraction cycle (*i*), quantified their differences, and averaged across all relaxation-contraction cycles within a session to yield a scalar measurement, as expressed in

$$a\left(p\right)=\frac{1}{n}\sum_{i=1}^{n}\left[a_{1}\left(p,i\right)-a_{2}\left(p,i\right)\right],$$

where n was the total number of relaxation-contraction cycles.

#### Rhythmicity.

The rhythmicity of the oscillation was defined as the ratio of the dominant oscillation relative to the overall deformation. Its estimation was done based on the power spectral density. We defined a narrow frequency range (2 cycles/min in both humans and rats) centered around the estimated dominant frequency of the oscillation. The rhythmicity was then quantified as the ratio of the power within the narrow frequency band relative to a broader frequency band (0.1 to 6 cycles/min in humans and 0.1 to 10 cycles/min in rats).

### Propagation Velocity

We quantified the propagation velocity along the greater curvature and the lesser curvature within the identified peristaltic pump. After noise reduction, we used a narrow band-pass filter centered around the local dominant frequency. It was implemented in the Fourier domain with a bandwidth of 2 cycles/min and a transition band of 0.15 cycles/min in both humans and rats. We first identified the most proximal vertices that had approximately zero-phase lags with the onset zone along the greater curvature (the lesser curvature), as the initiation of the gastric peristalsis along the greater curvature (the lesser curvature). Then for every two different vertices along the greater curvature (or the lesser curvature), we quantified their geodesic distance (*d*) on the time-averaged stomach surface and phase shift (ϕ) on their time series of displacements, and calculated the velocity (*v*) as

$$v=\frac{2\pi df}{\phi },$$

where *f* was the identified local dominant frequency of the oscillation. The phase shift (ϕ) between two time series of displacements was calculated by Fourier transform.

### Phase Coordination

The phase coordination map was defined as the averaged relative phase differences to manually selected points along the greater curvature (3 seeds in humans and 2 seeds in rats) and the lesser curvature (3 seeds in humans and 2 seeds in rats) within the peristaltic pump. These seeds were chosen if their phase differences were approximately zero relative to the onset zone. To estimate the instantaneous phases and phase differences between vertices, we filtered the displacements with a narrow band-pass filter, as in the estimation of the propagation velocity, followed by the Hilbert transform. The instantaneous phase was expressed as a complex vector. The relative phase difference per vertex was then estimated by quantifying its differences relative to selected seeds and averaging across time and expressed in radians.

### Coordination Index

The coordination index within the peristaltic pump was based on PLV defined as

$$\left|\frac{1}{T}\overset{T}{\underset{t=1}{\sum }}e^{j{\Delta \varphi }_{mn}(t)}\right|,$$

where Δφ*_mn_*(*t*) was the instantaneous phase difference between a particular vertex (*p_n_*) and a particular seed (*p_m_*) at time *t*. Note that the instantaneous phase difference was estimated similarly as in estimating phase coordination. We calculated the PLVs per vertex relative to 20 randomly selected seeds and averaged them across all seeds (total number of seeds *M*) and vertices (total number of vertices *N*) to yield the final coordination index as a scalar value, as expressed in

$$\frac{1}{MN}\overset{M}{\underset{m=1}{\sum }}\overset{N}{\underset{n=1}{\sum }}\left|\frac{1}{T}\overset{T}{\underset{t=1}{\sum }}e^{j{\Delta \varphi }_{mn}(t)}\right|.$$
